# Optimization of selection contribution and mate allocations in monoecious tree breeding populations

**DOI:** 10.1186/1471-2156-10-70

**Published:** 2009-11-06

**Authors:** Jon Hallander, Patrik Waldmann

**Affiliations:** 1 Department of Forest Genetics and Plant Physiology, Swedish University of Agricultural Sciences, SE-901 83 Umeå, Sweden

## Abstract

**Background:**

The combination of optimized contribution dynamic selection and various mating schemes was investigated over seven generations for a typical tree breeding scenario. The allocation of mates was optimized using a simulated annealing algorithm for various object functions including random mating (RM), positive assortative mating (PAM) and minimization of pair-wise coancestry between mates (MCM) all combined with minimization of variance in family size and coancestry. The present study considered two levels of heritability (0.05 and 0.25), two restrictions on relatedness (group coancestry; 1 and 2%) and two maximum permissible numbers of crosses in each generation (100 and 400). The infinitesimal genetic model was used to simulate the genetic architecture of the trait that was the subject of selection. A framework of the long term genetic contribution of ancestors was used to examine the impacts of the mating schemes on population parameters.

**Results:**

MCM schemes produced on average, an increased rate of genetic gain in the breeding population, although the difference between schemes was small but significant after seven generations (up to 7.1% more than obtained with RM). In addition, MCM reduced the level of inbreeding by as much as 37% compared with RM, although the rate of inbreeding was similar after three generations of selection. PAM schemes yielded levels of genetic gain similar to those produced by RM, but the increase in the level of inbreeding was substantial (up to 43%).

**Conclusion:**

The main reason why MCM schemes yielded higher genetic gains was the improvement in managing the long term genetic contribution of founders in the population; this was achieved by connecting unrelated families. In addition, the accumulation of inbreeding was reduced by MCM schemes since the variance in long term genetic contributions of founders was smaller than in the other schemes. Consequently, by combining an MCM scheme with an algorithm that optimizes contributions of the selected individuals, a higher long term response is obtained while reducing the risk within the breeding program.

## Background

The main goal of most breeding programs is to increase genetic merit while restricting the level of relatedness in the breeding population. If too many highly ranked candidates are selected, the genetic variance will be quickly reduced, thus compromising the long term response to selection and increasing the risk that individuals will suffer from inbreeding depression. It is, therefore, important to restrict relatedness within the population so that there is a healthy balance between genetic improvement and genetic variability. Hence, in breeding theory, much attention has been paid to developing selection methods to improve selection responses. [1] introduced the optimum contribution (OC) method for maximizing the selection differential at a predefined rate of inbreeding in the breeding population. OC is a dynamic constrained quadratic optimisation method that simultaneously selects the number of candidates and their respective contribution to the breeding population of the next generation. In comparative studies between OC and truncation selection, the former produces increased genetic merit at the same level of inbreeding, or a decreased level of inbreeding at the same level of genetic improvement; this has been demonstrated in simulations [1-3], using a deterministic approach [4] and in analyses of real data, e.g. [5-7]. [8] demonstrated how the OC method could be applied to tree pedigrees by using simulations to evaluate different long term breeding schemes. [9] examined the maximum reduction in coancestry at a specified level of genetic improvement in *Eucalyptus globulus*, whilst [10] assessed the increase in genetic gain achieved using the OC method in comparison with standard restricted selection in *Pinus sylvestris*. These studies are, to our knowledge, the only applications of quadratic optimisation selection in the field of tree breeding.

In general, the impact of mating schemes on genetic parameters has received less attention than the effect of the optimum contribution method. The effect on selection response seems to depend on the combination of methods used for selecting and allocating mates to create the next breeding population. For example, [11] and [12] found only a small difference in selection response that was attributable to minimum coancestry mating (MCM) compared with random mating (RM) in combination with truncation selection. However, [13] and [14] obtained a large improvement in genetic merit for an MCM strategy compared with random mating, when used together with a quadratic optimization selection method (i.e. OC). They argued that the MCM scheme avoids extreme relationships, e.g. full-sib matings, by connecting unrelated families. A population structure with less extreme relationships will improve the OC selection of candidates to contribute to the next generation, since the relationships between individuals with a high estimated breeding value (EBV) will be reduced. It has been demonstrated that the MCM strategy is particularly beneficial when the population is small, has discrete generations and the restriction on the increase in inbreeding is stringent [13,15]; this is often the case in tree breeding programs, e.g. [16]. Consequently, MCM could be a feasible option when choosing a crossing strategy to be used in a tree breeding program.

In forest tree breeding, most studies on the effect of mating schemes have compared positive assortative mating (PAM) and RM (e.g. [16-19]). The idea underlying PAM is to mate the best ranked trees with each other so that the between-family additive genetic variance of the population is increased [20]. As a result, selecting an elite part of the population could enhance the genetic merit further, i.e. selection for the deployment population [16-19]. None of the aforementioned studies used optimized dynamic selection methods; they used static selection methods where equal numbers of trees were selected from each generation irrespective of the pedigree of the total breeding population. [8] compared MCM to RM in combination with OC selection and found that MCM delayed inbreeding for one generation, but eventually the level of inbreeding reach the same level for MCM and RM. Similar conclusions have been reached by [13] and [21]. However, [8] did not compare differences in genetic improvement between different mating schemes under conditions where there was the same increase in relatedness. Furthermore, they only compared MCM in combination with minimization of variance in family size to RM. It is, therefore, necessary to investigate further the effect of different mating strategies on selection parameters when an OC algorithm is used in a tree breeding context.

Recently, it has been demonstrated that, when using quadratic optimization methods like the OC algorithm, the selective advantage is a function of the Mendelian sampling terms rather than the EBVs [22,23]. The breeding value of an individual can be broken down into three components [24]: (1) half of the EBV of the male parent; (2) half of the EBV of the female parent; and (3) the Mendelian sampling term, which is the aggregate deviation arising from sampling the segregation of alleles within the male parent and within the female parent. Simulations have shown that more accurate estimates of the Mendelian sampling term will lead to greater long term genetic gain without affecting the increase in inbreeding [22,23]. Improved accuracy of the Mendelian sampling term can be achieved by using an individual's phenotypic record or progeny information, as well as by development of more efficient algorithms for its estimation, for example [23].

The goal of the current study was to investigate how different mating strategies influence selection response and the accumulation of inbreeding when using OC selection within a typical forest tree breeding scenario. Comparisons were made between simulated populations over seven generations of selection. The simulated pedigrees were typical of those in tree breeding and the trees were assumed to be monoecious. The mating schemes were derived using a simulated annealing algorithm and objective functions were tested. The following mating strategies were evaluated: (1) RM with no constraints on mating relatives; (2) PAM with no constraints on mating of relatives but controls on the full-sib family size; (3) PAMCM: PAM combined with minimum variance in coancestry; (4) MCM1: regular MCM with no additional constraints; (5) MCM2: MCM combined with minimizing variance in family size; (6) MCM3: MCM combined with minimizing family size and finally; (7) MCM4: a combination of minimizing the variances in both coancestry and family size. We used stochastic Monte Carlo simulation procedures to obtain parameter estimates and genetic evaluations are performed using the individual tree model in a restricted maximum likelihood (REML) framework. Moreover, the theory of long term genetic contribution (i.e. based on regression of Mendelian sampling terms over generations) was used to predict any departure from the theoretical maximum limit of genetic gain.

## Results

All mating schemes resulted in similar levels of group coancestry for each generation, and these were slightly lower than the pre-defined levels. Tables 1 and 2 show summary statistics of Monte Carlo (MC) simulations for all schemes considered at the two pre-defined levels of coancestry and heritability (ΔC = 1; 2% and h^2 ^= 0.05; 0.25) for a maximum of 100 permissible crosses. The data in Table 3 represents the results when the maximum number of crosses between parent trees was set to 400; here we wanted to examine the effect of mating scheme on selection parameters when the number of crosses was large. Table 4 shows results from the regression analysis of the long term genetic contribution on their estimated Mendelian sampling term, which examines the efficiency of the mating schemes in terms of deviation from theoretical upper limit of genetic gain.

**Table 1:** Impact of mating schemes on selection parameters when ΔC = 1% and N_max _= 100

	**h^2^**	**F_7_**	**G_7_**	**∑r^2^/4**	**ΔV_A_**	**N_sel_**	**N_cro_**	**N_ful_**	***α*_7_**
RM	0.25	0.0589	6.65	0.00587	-5.0	69.1	94.3	53.1	-0.000995

PAM	0.25	0.0775	6.61	0.00599	3.2	68.1	87.9	57.0	0.0223

PAMCM	0.25	0.0780	6.36	0.00611	-3.5	67.8	86.7	57.8	0.0249

MCM1	0.25	0.0379	6.58	0.00589	-6.3	68.6	89.9	55.8	-0.0228

MCM2	0.25	0.0396	6.56	0.00579	-10.8	70.3	98.6	50.7	-0.0218

MCM3	0.25	0.0416	6.85	0.00585	-9.4	70.9	100.0	50.0	-0.0191

MCM4	0.25	0.0527	6.69	0.00600	-0.6	70.4	100	50	-0.00731

RM	0.05	0.0613	5.62	0.00631	-7.0	77.6	96.3	51.9	0.00128

PAM	0.05	0.0879	5.56	0.00660	3.7	76.2	91.4	54.8	0.0345

PAMCM	0.05	0.0871	5.48	0.00668	9.3	75.3	90.1	55.6	0.0361

MCM1	0.05	0.0389	5.72	0.00620	-4.0	76.1	92.8	53.9	-0.0228

MCM2	0.05	0.0399	6.01	0.00629	-4.7	78.3	98.9	50.5	-0.0223

MCM3	0.05	0.0418	5.91	0.00623	-4.9	78.3	100.0	50.0	-0.0198

MCM4	0.05	0.0536	5.81	0.00637	0.2	78.4	100	50	-0.00723

Investigated parameters were: accumulated inbreeding (F_7_), accumulated genetic merit (G_7_), sum of squared long term genetic contributions (∑r^2^/4), difference in additive genetic variance component as a percentage (ΔV_A_), average number of selected trees per generation (N_sel_), average number of crosses per generation (N_cro_), average number of full-sibs/family in each generation (N_ful_), amount of deviation from Hardy-Weinberg equilibrium (*α*_7_) at generation seven. Standard errors ranged between 0.0001 and 0.0016 for F_7_, 0.08 and 0.09 for G_7_, 0.00004 and 0.00024 for ∑r^2^/4.

**Table 2:** Impact of mating schemes on selection parameters when ΔC = 2%, N_max _= 100 and h^2 ^= 0.05

	**F_7_**	**G_7_**	**∑r^2^/4**	**ΔV_A_**	**N_sel_**	**N_cro_**	**N_ful_**	***α*_7_**
RM	0.124	7.07	0.0127	-9.0	44	83.5	60.0	0.00247

PAM	0.156	7.14	0.0129	7.8	42.5	74.8	67.1	0.0441

PAMCM	0.157	6.95	0.0127	5.2	41.6	70.3	71.5	0.0438

MCM1	0.0916	6.96	0.0113	-0.6	41.7	70.4	72.2	-0.0334

MCM2	0.0959	7.39	0.0119	-9.0	46.2	94.8	52.8	-0.0307

MCM3	0.105	7.51	0.0118	-4.8	46.3	100.0	50.0	-0.0206

MCM4	0.119	7.57	0.0121	7.3	46.3	99.4	50.3	-0.00639

Investigated parameters were: accumulated inbreeding (F_7_), accumulated genetic merit (G_7_), sum of squared long term genetic contributions of founders (∑r^2^/4), difference in additive genetic variance component in percentage (ΔV_A_), average number of selected trees per generation (N_sel_), average number of crosses per generation (N_cro_), average number of full-sibs/family in each generation (N_ful_), deviation from H-W equilibrium (*α*_7_) at generation seven. Standard errors ranged between 0.0004 and 0.0030 for F_7_, 0.11 and 0.13 for G_7_, 0.0001 and 0.0005 for ∑r^2^/4.

**Table 3:** Impact of mating schemes on selection parameters when ΔC = 1%, N_max _= 400 and h^2 ^= 0.05

	**F**	**G**	**∑r^2^/4**	**ΔV_A_**	**N_sel_**	**N_cro_**	**N_ful_**	***α*_7_**
RM	0.0628	7.22	0.00690	-12.5	112.1	338.1	14.8	0.000736

PAM	0.0830	6.87	0.00700	-2.4	108.1	279.9	17.9	0.0261

PAMCM	0.0837	7.13	0.00707	0.1	107.9	275.2	18.3	0.0251

MCM1	0.0409	7.15	0.00673	-6.8	108.8	302.9	16.7	-0.0223

MCM2	0.0430	7.24	0.00676	-9.5	115.3	359.1	14.0	-0.0212

MCM3	0.0471	7.29	0.00685	-7.5	116.9	400.0	12.5	-0.0165

MCM4	0.0590	7.32	0.00689	-10.4	118.9	396.5	12.6	-0.00374

Investigated parameters were: accumulated inbreeding (F), accumulated genetic merit (G), sum of squared long term genetic contributions of founders (∑r^2^/4), reduction in additive genetic variance component (ΔV_A_), average number of selected trees per generation (N_sel_), average number of crosses per generation (N_cro_), average number of full-sibs/family in each generation (N_ful_), deviation from H-W equilibrium (*α*_7_) at generation seven. Standard errors ranged between 0.0002 and 0.0014 for F_7_, 0.07 and 0.10 for G_7_, 0.00005 and 0.00008 for ∑r^2^/4.

**Table 4:** Residual variance of linear regression

	**ΔC[%]**	**h^2^**	***σ*_e_^2^[10^-4^]**
RM	1	0.05	3.81

PAMCM	1	0.05	3.98

MCM 2	1	0.05	3.76

RM	1	0.25	3.41

PAMCM	1	0.25	3.53

MCM2	1	0.25	3.25

RM	2	0.05	14.61

PAMCM	2	0.05	14.82

MCM2	2	0.05	13.68

RM	2	0.25	11.94

PAMCM	2	0.25	11.68

MCM2	2	0.25	11.30

Relationship between long term genetic contribution of founders (r) and Mendelian sampling term of the founders (a_est_) estimated at generation 7 when N_max _= 100 for schemes RM, PAMCM and MCM2.

### Genetic improvement

**Figure 1:**
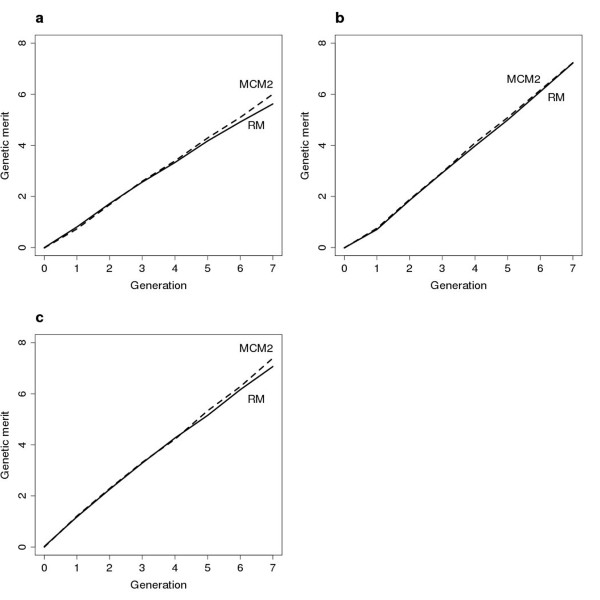
**Selection response**. Accumulated additive genetic merit in the breeding population when h^2 ^= 0.05 for RM and MCM2 (**a**) N_max _= 100, ΔC = 1%; (**b**) N_max _= 400, ΔC = 1%; (**c**) N_max _= 100, ΔC = 2%.

A less stringent restriction on coancestry resulted in higher genetic merit at generation seven (G_7_), in comparison with scenarios with very stringent restrictions (Figure 1; Tables 1; 2). In addition, the response to selection was higher when h^2 ^= 0.25 in comparison with h^2 ^= 0.05 due to the higher precision of EBVs. The overall benefit of MCM on G_7 _relative to RM was somewhat better when h^2 ^was small, although far from all MCM schemes produced any improved G_7_. MCM2, 3 and 4 always resulted in higher levels of G_7 _in comparison with RM, with the improvement ranging from 2 to 7%. The only exception was when ΔC was severely restricted and when h^2 ^was large, in which case RM resulted in similar levels of genetic merit in comparison with those obtained using MCM schemes (i.e. no significant difference was achieved in G_7_). The greatest impact of non random mating on G_7 _was for ΔC = 2% and h^2 ^= 0.05, resulting in 6.2% and 7.1% increased merit for MCM3 and MCM4, respectively. MCM1 always produced less gain than that achieved by MCM2-4, suggesting that the latter schemes produce a better population structure (i.e. they connect more families). This finding is supported by the average number of crosses for each scheme; MCM 2, 3 and 4 produced highest number of crosses in all scenarios (Tables 1, 2 and 3).

Both PAM and PAMCM resulted in similar levels of G_7 _to those produced by the RM scheme. There was no obvious benefit of avoiding extreme relationships in terms of accumulated gain because PAMCM did not increase G_7 _compared to RM. In fact, when h^2 ^was high and when there was severe restriction of ΔC, a considerably lower level of G_7 _was attained (4.4% lower than the G_7 _value attained by RM). PAM produced a somewhat higher G_7 _when ΔC = 2%, although the difference was not large.

The level of genetic merit increased for all schemes when the maximum number of permissible matings (N_max_) increased from 100 to 400 (Tables 1; 3; Figure 1). However, with the exception of PAM, which achieved somewhat lower merit, there was no significant difference in genetic merit at generation seven for RM than for the other schemes. Hence, if N_max _is increased, the relative importance of effective mating schemes on improvement in gain is reduced. In general, when N_max _= 400, the between-family selection intensity increased and, since the size of the full-sib families was rather large in both scenarios, the within-family selection intensity did not have a great effect on the outcome in G_7_. In addition, conversion of the mating proportions from the OC algorithm into the number of crosses for large N_max _would result in a better approximation to the optimal solution. Furthermore, Figure 1 shows that the rate of gain (ΔG) varied between the schemes, where MCM2 seems to have the highest ΔG for N_max _= 100 at later stages of the breeding program (generations 5 and 6). This suggests that MCM2 would increase genetic gain more than RM for selection schemes continuing beyond seven generations.

### Accumulated level of inbreeding

In general, the level of inbreeding was slightly higher for h^2 ^= 0.05 than for h^2 ^= 0.25 (Table 1). One possible explanation is that if the level of heritability is very low, the best linear unbiased predictor (BLUP) analysis takes more family information into account and then the OC algorithm selects more trees. When mating proportions of the selected trees are transformed into the number of crosses, the trees that make limited contributions can be discarded in mate allocations if h^2 ^is 0.05. MCM schemes were more efficient in reducing F_7 _in comparison with RM when ΔC was lower (i.e. more with rigid constraints). MCM1 yielded the lowest F_7 _of all schemes, reducing F_7 _by 26-37% in comparison with corresponding levels produced by RM. F_7 _was always lower for MCM1 than for MCM2, probably as a result of the extra restriction on variation in family size in MCM2, thus allocating mates that were least related. Moreover, compared with RM, MCM schemes always gave a lower sum of squared long term genetic contributions of the founders, suggesting better management of the founder contributions to descendants.

**Figure 2:**
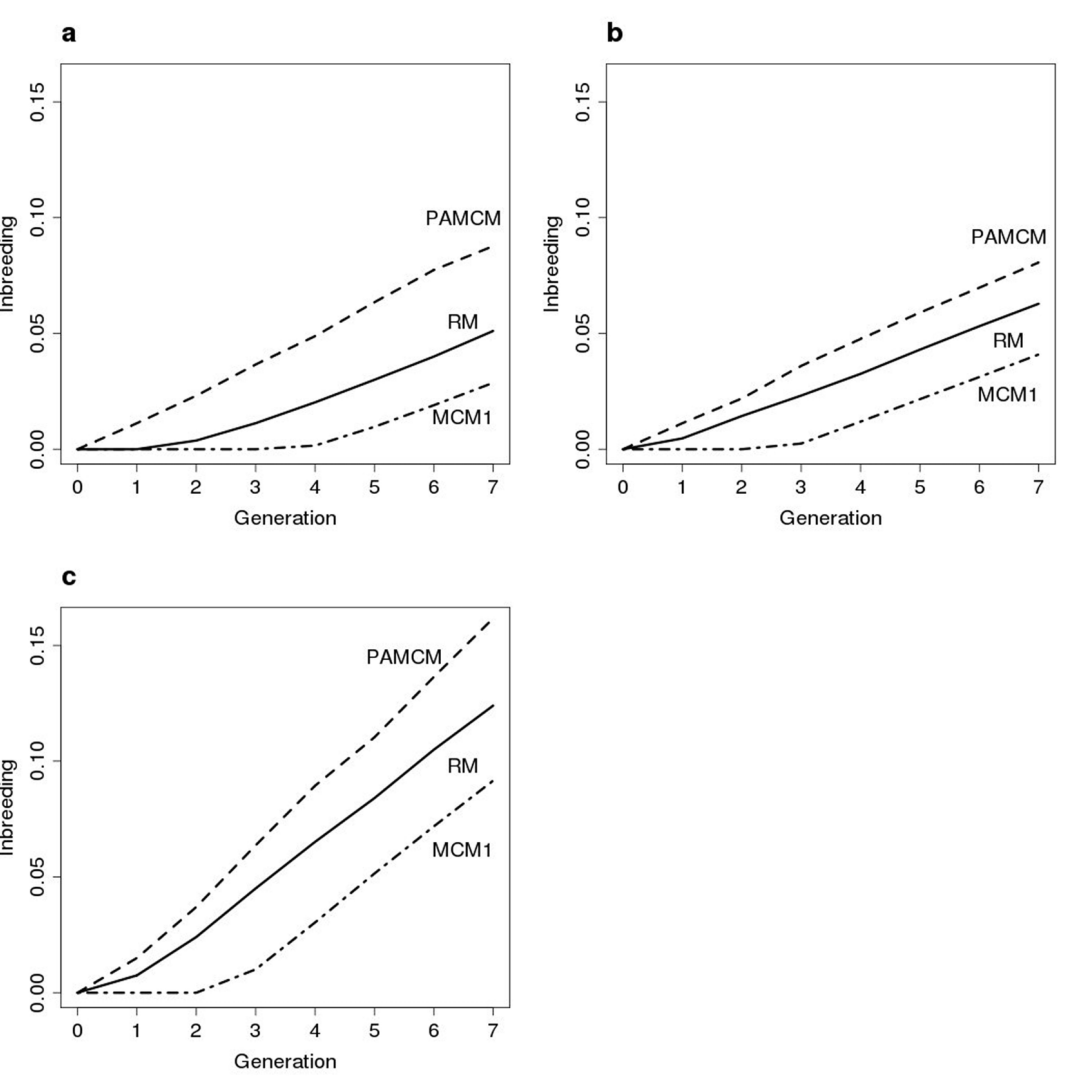
**Inbreeding**. Accumulated inbreeding in the breeding population when h^2 ^= 0.05 (a) N_max _= 100, ΔC = 1%; (**b**) N_max _= 400, ΔC = 1%; (**c**) N_max _= 100, ΔC = 2%.

There was a two generation delay in inbreeding in MCM1 in comparison with RM and PAM (Figure 2). The reason for such a result has been discussed in several studies, i.e. [8,11,21]. However, after the initial differences in inbreeding, the rate was similar between all schemes. The level of inbreeding for PAM was higher than the levels reported for RM, which agrees well with conclusions from similar studies, e.g. [16]. The F_7 _level was between 26 and 43% higher than corresponding levels obtained by RM. The most likely cause for this phenomenon may be a higher frequency of matings between related trees (i.e. more full-sib matings), particularly at low levels of h^2^. The high variance in the long term genetic contribution of founder trees and the strong positive deviation from ideal *α*_7 _is in accord with the high levels of inbreeding for PAM.

Perhaps not very intuitively, PAMCM always resulted in a high F_7_, between 27-42% more than corresponding levels for RM (Tables 1, 2, 3; Figure 2). The explanation can partly be seen in the large variance of founder contributions, suggesting that a few founders contribute a great deal and, consequently, the rate of inbreeding increases [24]. In addition, relatives are mated to a greater extent in PAMCM, which results in a large positive deviation from Hardy-Weinberg equilibrium (*α*_7_) compared to all the other schemes (except PAM). It seems probable that more crosses between half-sibs are allocated using PAMCM, due to the correlation in EBV between half-sibs combined with the relatively high number of crosses performed. As a result, more half-sibs (or other similar levels of relatedness) are crossed, particularly at low h^2^, because crosses between full-sibs are avoided (Tables 1, 2, 3).

When N_max _was set to 400, the level of accumulated inbreeding increased slightly in comparison with N_max _= 100 for all schemes (Tables 1; 3), with the exception of PAM and PAMCM. One possible reason is that trees that make a high contribution are involved in more matings if N_max _is higher, thus increasing the proportion of genes transmitted to the next generation. This conclusion is supported by the greater sum of squared genetic long term contributions of founders when N_max _is 400 compared to when it is 100. For PAM and PAMCM the lower level of *α*_7 _at N_max _= 400 suggests that the deviation from H-W equilibrium is less than for N_max _= 100, which would lead to a reduction in F_7_.

### Number of trees selected and crosses performed

In general, more selections and crosses were made when h^2 ^= 0.05 for all schemes than when h^2 ^= 0.25. The reason for this difference in number of selections might be that more family information is taken into account in the BLUP evaluations, which create higher correlations of EBVs between relatives. Consequently, OC selected more trees to reach the pre-defined level of coancestry when h^2 ^was low. For PAM, this difference was not very large in comparison with the difference for the MCM schemes, suggesting that the latter schemes performed better at low levels of h^2^. In addition, a stringent constraint on ΔC resulted in a larger number of both selections and matings at the same level of h^2 ^(Tables 1 and 2)

Since MCM3 allocated mates in order to minimize family size, the number of crosses performed was highest in comparison with all the other schemes. The difference in number of crosses performed between the schemes was most pronounced at ΔC = 2% (Table 2). Including a large number of families produces a better family structure (i.e. connects more families) and increases the possibility that the OC algorithm will select trees to contribute to the next generation within the restrictions on coancestry (i.e. increasing the between-family selection intensity). Clearly, the schemes that produce the highest levels of genetic gain also involve both a larger number of selections and more crosses. However, all schemes produced a very similar average number of selections, particularly when ΔC was small (Tables 1 and 3).

### Development of additive genetic variance components

**Figure 3:**
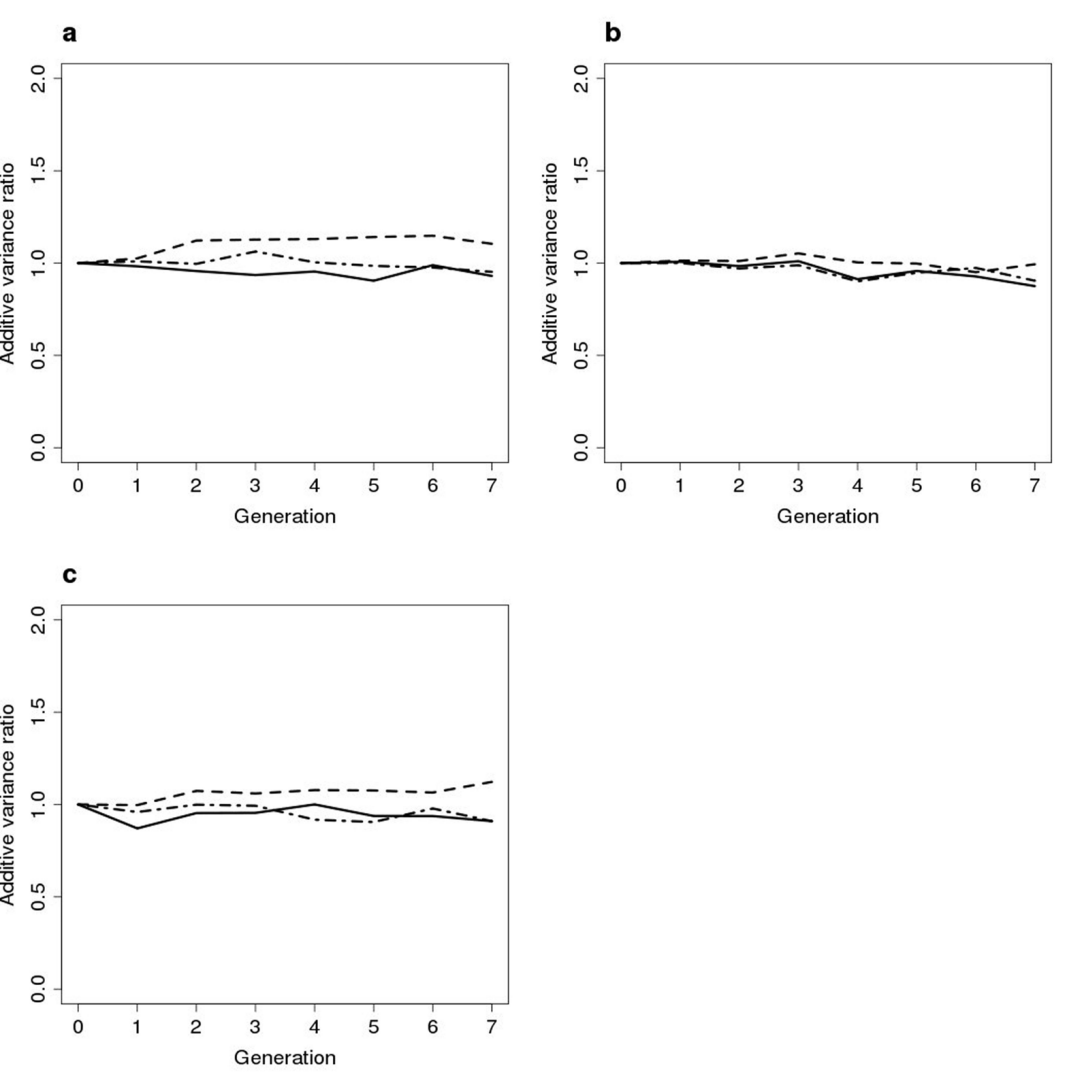
**Additive genetic variance**. Development of the additive genetic variance component in the breeding population when h^2 ^= 0.05, solid line: RM, dashed line: PAMCM and dashed-dotted line: MCM2 (**a**) N_max _= 100, ΔC = 1%; (**b**) N_max _= 400, ΔC = 1%; (**c**) N_max _= 100, ΔC = 2%.

Table 1, 2, 3 and Figure 3 demonstrate the reduction in the additive genetic variance component (V_A_) in the breeding population over seven generations of selection. We only show the additive genetic variance because non-additive genetic variance was assumed to be absent. No general difference in the trajectories of V_A _was detectable between mating schemes, apart from a slight reduction in most schemes that could be a result of either the Bulmer effect [25] or the build up of inbreeding. This result suggests that if OC selection is applied to the breeding population, the choice of mating scheme does not greatly affect the development of the additive genetic variance. On the other hand, PAM and PAMCM resulted in the lowest reduction in V_A_, sometimes even an increased V_A _was observed after seven generations of selection. Moreover, when h^2 ^was low, more trees were selected on average, thus slowing down the reduction in V_A_.

The impact on the additive variance of setting N_max _to 400 is shown in Table 3 and Figure 3b. In all cases, V_A _was reduced more when N_max _= 400 in comparison with N_max _= 100. This may be because of the increased sum of squared long term genetic contributions or the increased level of inbreeding, reducing the within-family additive variance component more compared to N_max _= 100 (Tables 1 and 3, Figure 3).

The mean squared error (MSE) over replicates (MC iterations) of estimated variance components varied between mating schemes. For V_A_, RM and MCM schemes resulted in approximately constant MSE over generations. PAM and PAMCM showed a similar pattern for the first five generations, but MSE increased during last two generations. For V_E_, no real trends were detectable, suggesting that accuracy of REML estimates of V_E _were unaffected by the number of generations in the pedigree. MSE results are presented in Additional file 1.

### Long term genetic contributions and selective advantage

**Figure 4:**
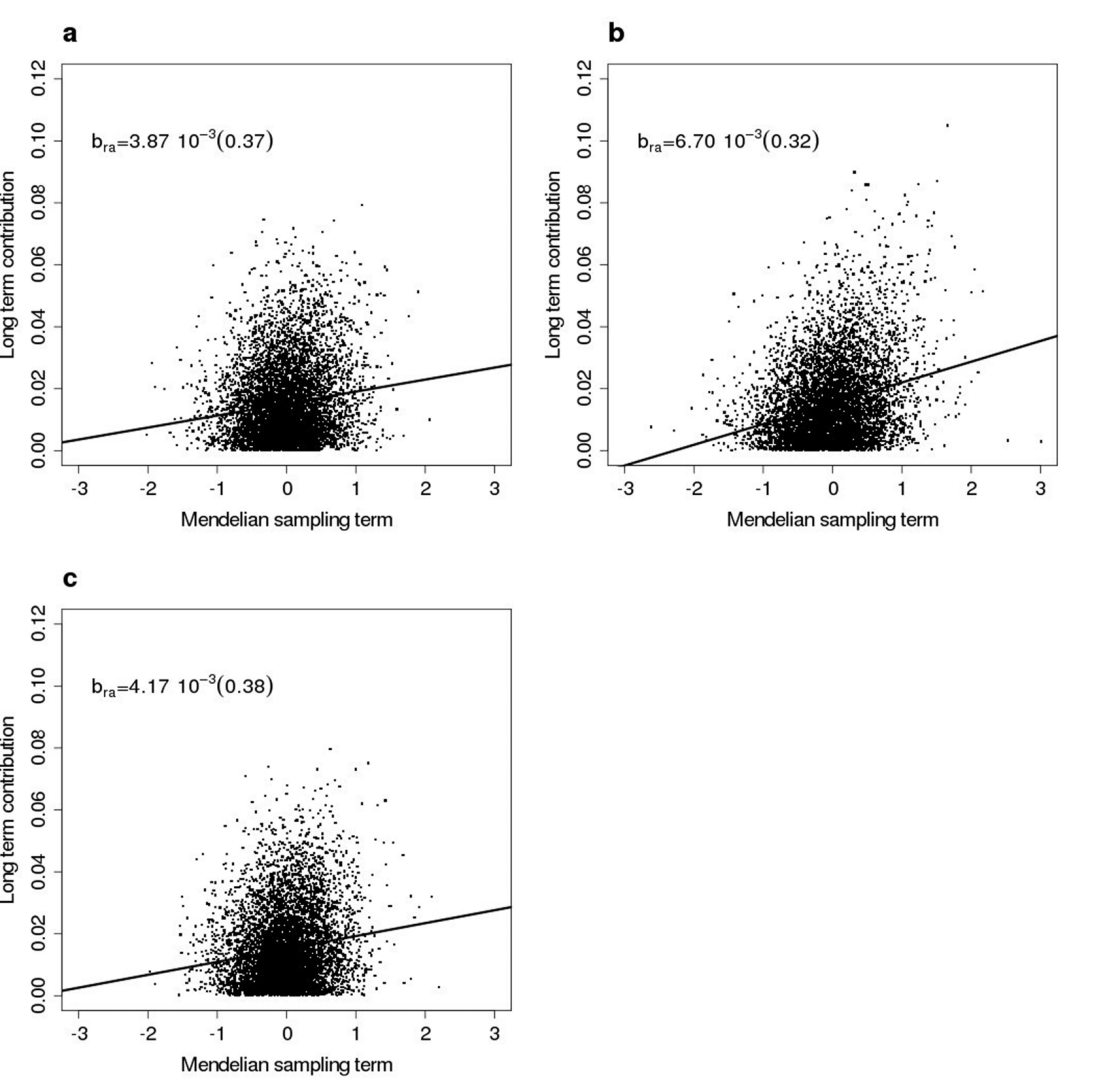
**Regression of long term contribution against Mendelian sampling term**. The association between long term genetic contribution and Mendelian sampling term of the founders estimated at generation 7 and the value of the regression coefficient, b_ra_; the standard error is given in parentheses. N_max _= 100, ΔC = 1% and h^2 ^= 0.05: (**a**) RM; (**b**) PAMCM; (**c**) MCM2.

Figure 4 demonstrates the influence of the estimated Mendelian sampling terms (*a*_*est*_), at generation 7, on the long term genetic contribution (*r*) of the founders. Table 4 lists the residual variance of the linear regression. It should be noted that *r *displayed non-zero variation over selection candidates at generation seven (i.e. for founder i:*Var*(*r*_*i*__1_, *r*_*i*__2_, ..., *r*_*iN*_) > 0, where N is the number of selection candidates in generation 7) in all scenarios, which could indicate a lack of convergence. Nevertheless, the results provide information on the efficiency of the different mating schemes (i.e. in managing the contribution of the founders). We have chosen to present the three most interesting schemes in terms of accumulated genetic merit, namely RM, PAMCM and MCM2. Clearly, the difference in residual variance () of the linear regression varied between the different mating schemes, indicating differences in departure from the optimal allocation of *r *on *a*_*est*_. In all scenarios, MCM2 resulted in lower  compared to the corresponding  obtained by RM and PAMCM. In addition, PAMCM always produced the highest  of the schemes considered. The difference in  can be seen in Figure 4, where some founders have a much larger *r *in comparison with the optimum (i.e. the regression line), this is particularly clear in Figure 4b. In Figure 4c the *r *values are distributed much more evenly around the line in comparison with their distribution in Figure 4b. The optimal allocation of *r *clearly depends on the level of the heritability (Table 4). The slopes of the regression lines are plotted in Figure 4, demonstrating that PAMCM produced the largest regression coefficient (*b*_*ra*_) of all schemes. This result implies that the contribution of selected trees became more variable for PAMCM compared with the other schemes and that a few trees make a very high contribution (see also Figure 4b). Consequently, a more equal long term contribution of founder trees facilitates the OC algorithm in terms of deviation from the ideal solution during selection decisions. The patterns found here would, however, be clearer if the variance in long term contributions of founders over descendants would be reduced further (i.e. by including more generations of selection).

## Discussion

Here we have demonstrated that different strategies for mate allocation result in different genetic parameters in a tree breeding scenario when optimized contribution selection (OC) was applied over seven generations. In the scenarios considered, the rate of increase in coancestry (ΔC) was restricted to either 1 or 2% for two different levels of heritability (0.05 and 0.25). We found that, in general, the minimum coancestry mating (MCM) schemes produced a higher level of genetic merit (G_7_) and lower level of inbreeding (F_7_) after seven generations of selection compared to equivalent results achieved by RM. Up to 7.1% increase in genetic merit was achieved by MCM2, MCM3, and MCM4 in comparison with corresponding results obtained by RM. On the other hand, MCM1 yielded the lowest level of accumulated inbreeding, with a maximum decrease in F_7 _of 37% compared with the equivalent estimate obtained through RM. The two mating schemes that used positive assortative mating combined with restrictions on variance in either family size or coancestry (PAM and PAMCM, respectively) resulted in similar levels of accumulated genetic merit but higher levels of inbreeding compared with RM. In addition, regressions of the long term genetic contribution (*r*) of founders on estimated Mendelian sampling terms (*a*_*est*_) showed that the minimum coancestry schemes resulted in lower residual variance and, therefore, less deviation from the ideal level of genetic gain. We also demonstrated that the MCM schemes resulted in lower sums of squared *r *for founders, suggesting that these schemes produce the lowest rate of inbreeding (ΔF) in the population. For estimates of *r*, we used a robust, deterministic approach that can handle large, complex pedigrees, as suggested by [26].

### Response to selection

There are several reasons why the MCM schemes produced a better response to selection. First, because of the larger number of families created, the between-family additive variance was greater; this can be exploited by the OC algorithm. This is probably one of the main effects that enhanced the selection response in comparison with RM, since the number of both selections and crosses performed using the MCM schemes was always higher. PAM and PAMCM produced a number of selections and levels of accumulated gain that were similar to RM. We also found that a larger number of crosses produced a better integer approximation to the optimal solution when converting the mating proportion suggested by the OC method into the actual number of matings. Second, in the framework of long term genetic contributions, MCM schemes showed least deviation from the theoretically attainable genetic merit in the population. The reason for this outcome is probably because MCM schemes result in better management (usage) of long term genetic contributions by founders when selecting trees and their mating proportions for each generation of selection. Hence, a more even contribution of trees and avoidance of mating between close relatives caused unrelated families to be connected to a greater extent, thus the OC algorithm can increase the selection differential. Third, a lower level of inbreeding in MCM schemes resulted in a lower reduction in Mendelian sampling variance (less within-family additive variance). Therefore, the level of within-family selection response will be diminished according to *R*_*w *_= *i*_*w*_*σ_Aw_h_w _*, where *σ*_*Aw *_is the within-family additive standard deviation, *i*_w _is the within-family selection intensity and *h*_W _is the square root of the within-family heritability. The relatively high level of inbreeding in the PAM and PAMCM schemes is probably one of the reasons that they yielded slightly lower levels of selection response than RM and MCM.

When the maximum number of created families in each generation was increased from 100 to 400, in general, increased levels of genetic merit were obtained. However, the relative differences in genetic merit between the mating schemes were reduced. The most likely reason is that management of the long term contribution of ancestors is more important in schemes where fewer full-sib families are created in each generation, since the OC algorithm provides a better opportunity for increasing the between-family selection intensity if the relationships between families are more equal. Consequently, if suitable mating strategies are utilized, such as the MCM schemes, the level of genetic gain will be enhanced in schemes where the number of crosses is few.

A simulation study of animal breeding programs [13] found that schemes that minimize coancestry yield an increased long term response of up to 22% in comparison with RM schemes after 20 generations of selection. Similar results were found by [14] in a study that allowed for selections over multiple generations. The greater differences in selection response between MCM and RM schemes reported in these studies, compared with our findings, suggest that it is even more favourable to employ an MCM scheme in long term breeding. In addition, [13] found that a strict restriction on the allowed rate of inbreeding (ΔF) favours MCM schemes in terms of accumulated genetic merit in comparison with that achieved by RM. However, with a less stringent restriction on ΔF, they achieved less difference in merit between MCM and RM. Our results contradict this finding since we obtained a greater difference in merit between MCM schemes and RM when the restriction placed on relatedness was less stringent. One reason may be that different levels of enhanced within-family selection response were obtained for the different mating schemes in our study as a result of the population structure (i.e. the size of full-sib families used).

When PAM has been combined with static selection methods, little improvement in response to selection in the breeding population has resulted, compared with RM, i.e. [17,19]. As a mating method, PAM is not designed to improve population structure, but instead tries to separate the population into several sub-lines. Initially, we simulated a strict PAM scheme without the additional restriction on family size. However, very few crosses were made and this severely restricted the OC algorithm in terms of accumulated genetic gain (results not shown). When we included minimization of variance in family size, enhanced levels of genetic gain were obtained. Moreover, for both the PAM and PAMCM schemes, we found higher levels of inbreeding, thus reducing the within-family additive genetic variance (Mendelian sampling term). In the short term (one or two generations), however, the impact of the population structure is low in terms of genetic improvement. It should be emphasised, that the purpose of PAM is to increase between-family additive variance, while the OC algorithm counteracts the effect of PAM by attempting to decrease the between-family variance. All MCM methods have the opposite effect, since they try to avoid matings between relatives as much as possible, leading to a better population structure because unrelated families are connected to a greater extent. Hence, by increasing the solution space (i.e. fewer related families to choose from), OC can benefit more from the resulting population structure.

### Inbreeding

The level of coancestry will inevitably rise when directional selection is applied to a closed breeding population. Consequently, inbreeding will also accumulate. By using MCM, the level of inbreeding can be delayed, but will eventually reach the same level as RM. The reason why MCM and RM will generate similar levels of inbreeding in the long term is that all MCM schemes produce greater negative deviation from Hardy-Weinberg equilibrium (see Results). Hence, the asymptotic level of inbreeding will be equal even though the level of inbreeding is lower in the short and medium terms considered here. [21] compared the performance of mating schemes for conservation purposes and suggested that minimum coancestry mating would produce higher levels of accumulated inbreeding after approximately 300 generations, i.e. well outside the range for most forest tree improvement scenarios.

### Genetic contributions of ancestors and pedigree development

We have shown that OC selection provides different departures from the ideal attainable genetic gain for the mating schemes implemented here with restrictions placed on coancestry. We draw this conclusion by examining the residual variance of the regression line of *r *on *a*_*est*_, corresponding to the part of *r *that does not contribute to the overall genetic gain in the breeding population [22]. The MCM strategy combined with minimization of the variance of family size yielded lower residual variances in all scenarios compared with RM and, consequently, resulted in a genetic gain that was closer to the optimal attainable gain. The difference in residual variance between the mating schemes is partly a result of the management of r in the population, which is improved by connecting unrelated families or controlling family size, amongst other things. As a result, the OC algorithm increases selection intensity between families. There are other factors that contribute to the variance around the regression line. [2] suggested that as more information about a pedigree becomes available (i.e. increased accuracy of estimates of both *r *and *a*), the contribution of each generation proposed by OC will also change (see also [27]). Since true breeding values are not known, deviation from the optimal solution will be a result of estimation errors, particular at lower levels of heritability. In addition, since the selection program as a whole is a multi-generation process, contributions cannot be obtained independently without changing the long term contributions of ancestors [22,27]. However, more work is needed to better understand the mechanisms behind the relationships between genetic gain and pedigree development in a quadratic index framework; for example, producing theoretical predictions of the attainable rate of gain that can be achieved by different mating designs and restrictions on relatedness.

### Practical considerations in tree breeding

[8] suggested that OC selection could be used for clone selection for deployment populations (e.g. seed orchards). OC should increase the genetic merit of the seeds obtained in the orchard compared with merit obtained from non-optimal selection methods. The genotypes selected for seed orchard use will be a subset of the genotypes included in the main breeding population. Therefore, a restriction on relatedness of the selected subset of clones is needed to maintain a reasonable level of genetic variability. Hence, by ensuring careful management of the long term genetic contributions of ancestors and by connecting unrelated families, the OC method would increase genetic gain in the deployment population by increasing selection intensity between families in the breeding population. In addition, it is important to take the additive genetic variance (V_A_) of the breeding population into consideration since a higher level of V_A _could be exploited [16]. We found no clear pattern when examining the trajectories of V_A _for the different mating schemes, although PAM and PAMCM yielded less reduction and sometimes even a slight increase in V_A _compared with the other schemes. On the other hand, PAM and PAMCM increased the level of inbreeding by up to 44% in comparison with RM and even more in comparison with the MCM schemes; this is not desirable in production populations. This difference between the mating schemes is important to remember.

The levels of heritability (0.05 and 0.25) used in the current study were based on levels reported in [10], which are representative for traits in breeding populations of conifer species. Typically, traits that correspond to volume production are important, such as diameter at breast height (DBH) and stem height (H), where DBH and H correspond to low and high levels of heritability.

It should be pointed out that the SA algorithm, for some of the mating schemes, was assigned to minimize an object function containing two separate terms or objectives (see Additional file 2 for all object functions used). As a result, this approach might give an unbalanced response on the distinct objectives, because it might favor the objective that is most variable across permutations. This issue needs to be further investigated in order to optimize multiple objectives more efficient using the SA algorithm in breeding situations.

## Conclusion

By using different mating schemes combined with optimum contribution selection, different levels of response to selection and accumulation of inbreeding were found in a typical tree breeding scenario. The differences in the parameters obtained between the mating schemes were most obvious when the number of controlled crosses in each generation was small. Minimum coancestry mating resulted in the greatest level of genetic gain, while the level of accumulated inbreeding was significantly lower in all scenarios. Positive assortative mating schemes yielded a similar level of genetic gain as random mating, although the level of accumulated inbreeding was significantly higher in all cases. Our findings are supported by the theory of long term genetic contributions.

## Methods

### Selection procedure

[10] presented a modification of the OC algorithm in [8], which was used to select individuals dynamically. The quadratic objective function *f*(**c**_**t**_) of the OC algorithm is obtained by introducing LaGrangian multipliers, *λ*_0 _and *λ*_1_, combined with the constraints on relatedness and on total contribution

(1)

where **c**_**t **_is a vector containing the mating proportion of the candidate trees in the breeding population at round t (' denotes the transpose of the vector), **A**_**t **_is the additive relationship matrix between candidate trees, **b**_**t **_is a vector containing the EBV of the candidate trees, **1 **is a vector of ones in all entries and C_t+1 _is the constraint on group coancestry in the population at generation t+1. The restriction on group coancestry holds if the increase between generations is small [1]. In most studies using OC selection, ΔF is used to restrict the selection of individuals in each generation rather than the increase in coancestry in the breeding population, i.e. [2,13]. However, we decided to restrict group coancestry, since it is not influenced directly by the mating scheme. Furthermore, we restricted the total mating contribution instead of the contributions from each sex, since most conifer forest tree species are monoecious (see details in [8]). In addition, in order to obtain an appropriate mating program, **c**_**t **_needs to be transformed into integer values; these are called contribution units (***ζ***_**t**_) [8]. ***ζ***_**t **_specify how many potential crosses (matings) each tree can participate in. Furthermore, the limit of the allowed maximum number of crosses each generation is set to N_max _= ∑_i_*ζ*_t, i_/2. Depending on the outcome of the mate allocation procedure (described in next section), the actual number of crosses performed (N_cro_) could then be less or equal to the allowed maximum number of crosses N_max_. Each cross between two parents resulted in one family where the size of the family (i.e. the number of full-sibs) was computed as dividing the total number of plants available each generation (5000) with N_cro_. Here, we used the following procedure to convert **c**_**t **_into ***ζ***_**t**_

1. multiply the contribution vector by twice the maximum number of crosses: 2 N_max_**c**_**t**_

2. round down 2 N_max _**c**_**t **_= ***ζ***_tmp _to the nearest integer below the actual value for each tree i, to obtain the temporary number of crosses summed over all trees ∑_i_***ζ***_tmp, i_/2 = N_tmp _(i.e. an integer value)

3. count the total number of families produced so far, N_tmp_

a. if N_tmp _< N_max_

i. increase the number of matings for the tree i with the highest deviation between the real and integer numbers by one (***ζ***_tmp, i _= ***ζ***_tmp, i _+ 1) and consequently increase N_tmp _by one

b. otherwise

**i**. exit the loop and set ***ζ***_**t **_= ***ζ***_tmp_

where N_tmp _and ***ζ***_tmp _are temporary variables.

### Mating optimization procedure

Optimization of the mating proportions selected individuals in each generation was performed using the simulated annealing (SA) approach of [8]. In each generation, the SA algorithm is used to obtain a mate allocation matrix, **X**, defining how mating between selected individuals should be performed in the breeding program. The order of **X **is *n *× *n*, where *n *is the number of selected parents. **X**(*i*, *j*) = 0 indicates that trees *i *and *j *are not mated and consequently, if **X**(*i*, *j*) > 0, trees *i *and *j *are mated. In addition, the number of non-zero elements in **X **is the actual number of crosses N_cro _while the sum of all non-zero elements in **X **is the maximum number of crosses N_max _(i.e. ∑_*i*_∑_*j*_**X**(*i*, *j*) = N_max_). The SA algorithm is a stochastic search algorithm that tries to locate a global minimum of a loss function [28]. The main advantage of using the algorithm is that it avoids getting stuck at a local minimum en route to a global minimum. Each iteration of the SA algorithm starts by defining the loss function, L(**X**), then two randomly chosen matings are rearranged using uniform random numbers (i.e. matings (*i*, *j*) and (*k*, *l*) are randomly chosen and are permuted into (*i*, *l*) and (*k*, *j*) so that **X **is permuted into **X***'*). A new loss function, L(**X***'*), is computed and compared with L(**X**). If L(**X***'*) < L(**X**), the new configuration is kept.

However, if L(**X***'*) > L(**X**), there is still a chance that the new configuration will be accepted, depending on the "temperature" of the system. As the number of iterations increases, the temperature decreases (i.e. the system cools down) and the probability of accepting a new configuration if L(**X***'*) > L(**X**) decreases. The probability of accepting a new state (*p*_*k*_) if L(**X***'*) > L(**X**) is

(2)

where *c*_*b *_is the Bolzmanns constant and *T*_*k *_is the temperature of the system [29]. Furthermore, since there is full control of the temperature, *c*_*b *_is set to *1*. The temperature decays according to *T*_*k *+ 1 _= *T*_*k *_(1 - *α*) where *α *is chosen according to the size and complexity of the optimization problem. In the current study, *α *was set to 0.01 and *T*_0 _to 1. Eventually, as the iterations proceed, *T*_*k *_becomes very low and no further changes occur; at this point, we obtain one solution to our optimization problem. The initial **X **was obtained by first ranking all trees according to their contribution and then assigning as many contribution units between the top ranked and the second ranked tree as possible, i.e. **X**(1, 2) = *ζ*_t, 2_, since *ζ*_t, 2 _≤ *ζ*_t, 1_. Then the first and third ranked trees are assigned contribution units and so on until all contribution units of the best ranked tree are assigned. By continuing this procedure, all contribution units are allocated between all selected trees, even though it might require adding an extra unit to *ζ*_i _of the penultimate ranked tree. This approximation will most probably have very little influence on the outcome of the simulation process. The SA algorithm has been used extensively for calculating optimal mating schemes in various breeding situations, e.g. [13,15,30].

### Mating strategies

The following mating strategies were compared:

RM - random mating with no constraints on the mating of relatives. L(**X**) was set to a constant which was kept throughout the iteration procedure, leading to all suggested mating changes being accepted. The total number of changes (iterations in the SA algorithm) was set to 30 000.

PAM - positive assortative mating based on EBV combined with minimization of variance in family size. Hence, L(**X**) consisted of two terms: one computed the difference in EBV between mating pairs while the other computed the variance in allocation of contribution units between trees. A strict PAM scheme with no constraints on the mating of relatives was implemented first (i.e. only the rank of parents was used), but this lead to very large family sizes, which severely restricted the OC algorithm.

PAMCM - PAM combined with minimization of coancestry variance (scheme MCM4), because it is very likely that close relatives will be mated in a strict PAM scheme. Estimated EBVs tend to be similar within families and therefore full-sibs are likely to be adjacent to each other, particularly at the lower level of heritability considered here (h^2 ^= 0.05). As a result, full-sibs will be mated more often in PAM than in a RM scenario; this could severely restrict the OC algorithm when selecting from all available candidate trees. Therefore, a combination of PAM and minimization of coancestry variance should lead to a more appropriate population structure, and resulting in an improved outcome for the OC algorithm in comparison with strict PAM. Here, L(**X**) contained the same term as in the PAM scheme (pairwise EBV between potential mates) but also minimizing variance in pairwise coancestry between mates.

MCM1 - standard minimum coancestry mating (MCM), which assigns mating pairs based on the lowest possible pairwise coancestry with no additional constraints. Hence, inbreeding in the offspring generation is minimized. Theoretically, this option should produce the lowest variance in long term contributions in comparison with all other schemes since the only aim of the scheme is to minimize pairwise coancestry between mates. Consequently, the increase in inbreeding of the population will be minimized [31].

MCM2 - To achieve a more even population structure, MCM was combined with minimization of variance in family size. By adding this feature to the standard MCM, the genetic merit of the breeding population should be enhanced when using a quadratic selection procedure, as demonstrated by [13]. L(**X**) comprised of one term minimizing coancestry between mates (see MCM1) and one term minimizing variance in family size (see PAM).

MCM3 - MCM combined with minimizing family size, which would behave in a similar manner to a factorial mating scheme. It has been predicted that this mating scheme will decrease inbreeding in animal breeding situations [32] and reduce the increase in relatedness in tree breeding [33]. Here, L(**X**) contained one term minimizing coancestry between mates (see MCM1) and one term minimizing the family size so that as many half-sib families were created as possible.

MCM4 - here, minimal variance in coancestry was used, since this option should avoid extreme matings (i.e. full-sib matings) and therefore, produce a better population structure. To improve the population structure further, we added minimization of variance in family size. Consequently, L(**X**) contained terms that minimized variance in both pairwise coancestry between mates (e.g. PAMCM) and in full-sib family size (e.g. PAM).

We ran the SA algorithm with different numbers of iterations and different decreases in temperature, depending on the ratio of accepted/non-accepted proposals for **X**. In one case (i.e. RM), simpler algorithms for computing the mating scheme may have been more convenient. Nonetheless, since we already had the SA algorithm implemented in the simulation program, we choose to utilize it as much as possible. This also facilitated comparison between mating schemes because they were all based on the same algorithm. Mathematical descriptions of the loss functions that define the mating schemes are presented in Additional file 2.

### Long term genetic contribution and Mendelian sampling term

[34] introduced the concept of long term genetic contribution, *r*_*i*_, which is the proportion of genes, inherited from ancestor *i *by a defined generation of descendants. For a non-random mating population, [31] proved that the increase in inbreeding, Δ*F*, is a function of the sum of squared long term genetic contributions from ancestors to descendants, , according to , where *α *is the departure from complete random mating (i.e. deviation from the Hardy-Weinberg equilibrium). Hence, to minimize the rate of inbreeding in the population, the sum of squared long term genetic contributions should be minimized. To estimate *r*_*i *_for all ancestors *i*, we used the deterministic approach proposed by [26]. Since we found a non-zero variation in *r*_*i *_over different descendants (i.e. the long term genetic contribution of founders did not converge), we used  where *r*_*ij *_is the long term contribution of ancestor *i *to a particular descendent *j *and *N *is the total number of offspring in the last generation (i.e., *N *= 5000). Here, we chose the founder population as ancestors when estimating .

In selection algorithms using quadratic indices, [31] defined the expected rate of genetic gain as a function of the long term genetic contributions of ancestors and their respective Mendelian sampling term (*a*)

(3)

Hence, (3) indicates that accumulated genetic gain in the breeding population depends on utilization of the Mendelian sampling term, which corresponds to each individuals' unique contribution to the gene pool. Another important property of (3) is that the rate of gain in the population is related to the pedigree (by means of *r*_*i*_), which is not apparent with the standard quantitative genetic formula for gain (i.e. the breeders equation [35]). A convenient mating scheme can, therefore, improve the rate of gain in the breeding population through better management of *r*. Moreover, [2] demonstrated that when using quadratic indices, the ideal solution is obtained when the long term genetic contributions of selection candidates are assigned in an exact linear fashion to the best available estimate of their Mendelian sampling term. The variation around the regression line of *r *on *a *would then correspond to the departure from the optimal solution (i.e. the maximum attainable ΔG given the constraint put on relatedness), which [22] used to prove that the selective advantage is a function of *a*. We used the following linear regression to determine the impact of the mating scheme on the allocation of *r *on *a *at generation 7:

(4)

where *b*_*ra *_corresponds to the regression coefficient, *a*_*est *_is the estimated Mendelian sampling term, *c *is the intercept and *e*_*i *_~*N*(*0*, *σ*_*e*_^2^) is the residual effect. Only trees having a positive contribution (i.e., selected trees) were included in the regression analysis. High values of *b*_*ra *_indicate a less equal contribution (utilization) of the selected individuals [2]. Typically, the value of *b*_*ra *_depends on the restrictions placed on relatedness and how the population structure is improved by the mating scheme.

### Simulated data

The infinitesimal genetic model [25] was used to simulate a tree breeding program over multiple generations. The initial population of 100 founders was assumed to be in HW-equilibrium, i.e. unselected and unrelated, where the true breeding value of founder *i *was generated from *N*(0, ) and the phenotypic value was created by adding a normally distributed environmental deviation to the genotypic value, sampled from *N*(0, ), where  and  corresponds to the additive genetic and environmental variances in the founder population. Initially,  was always 1 while  varied depending on the level of heritability used in the simulation. Two different levels of heritability were evaluated (0.05 and 0.25), and two constraints on ΔC were tested (1 and 2%). No systematic environmental or non-additive genetic effects were simulated. In addition, each founder were crossed with two other founders according to a double-pair mating design where each full-sib family contained 50 full-sibs, resulting in 5000 selection candidates in total. Equal population size was maintained throughout the simulation. OC selection was then applied to the breeding population over seven discrete generations; genetic and population parameters were calculated and stored for each generation. The simulation started at generation zero where the founders were generated and finished after generation seven. Two different maximum numbers of crosses (i.e. number of families) per generation were tested in order to examine how the different population structures affected the selection parameters (100 and 400). Since the OC algorithm required inverting the additive relationship matrix of available selection candidates, we chose to restrict the number of candidates from each full-sib family according to their EBV so that the full-sibs having the highest EBV were available for selection. The number of restrictions on available full-sibs depended on the constraint on Δ*C *and the available mating scheme under evaluation in the current simulation, but varied typically between 5 and 10. To further improve the speed of the OC algorithm, we implemented the method suggested by [36]. See [9] for an alternative selection method based on the simulated annealing approach that avoids inversion of the relationship matrix. The candidate trees were then mated according to **X**, obtained from the SA algorithm, creating new selection candidates. During generation t, the additive values of the offspring were sampled from , where  is a vector containing the average true breeding values of the parents in the order 5000 × 1 (i.e. one element for each candidate tree), and **A**_t _is the additive relationship matrix between candidates in the order 5000 × 5000. EBV and genetic variance components were estimated for each generation using the individual tree model [37,38]. The software used in the genetic evaluation procedure was ASReml [39]. In addition, the deviation from H-W equilibrium at generation *t*, *α*_*t*_, was computed using Wright's F-statistics [40]

(5)

where *k*_*t *_is the average pairwise coancestry and *F*_*t *_is the average inbreeding coefficient in the selected population. *F*_*t *_and *k*_*t *_were obtained from the additive relationship matrix at generation *t*. After completing seven generations of selection, the long term genetic contributions of the founders were estimated by using the algorithm suggested by [26] and all additive effects (i.e. true breeding values) were stored. In total, 100 replicates were generated and median values of the parameters of interest were calculated.

## Authors' contributions

JH produced and developed the computer code, ran the analysis and wrote the manuscript. PW planned and organized the study. Both authors have examined and approved the final version of the manuscript.

## Supplementary Material

Additional file 1 **Mean squared error of REML estimates over replicates**. This section includes a table showing the mean squared error of REML estimates of the variance components (that is, V_A _and V_E_) over replicates. Click here for file

Additional file 2 **Object functions for the various mating schemes**. This section contains a full list of object functions used throughout the study. Click here for file
