# Supplementary material for: Optimization of selection contribution and mate allocations in monoecious tree breeding populations
Source: BMC Genet. 2009 Nov 6;10:70. doi: 10.1186/1471-2156-10-70 (PMC2776599; doi:10.1186/1471-2156-10-70)
Supplement: Additional file 1 — Mean squared error of REML estimates over replicates. This section includes a table showing the mean squared error of REML estimates of the variance components (that is, VA and VE) over replicates. [file 1471-2156-10-70-S1.pdf]

Mean squared error of REML estimates over replicates

Additional table 1: mean squared error (MSE) over replicates of REML estimates of variance components for the various mating strategies for h^2 = 0.05, N_{max} = 100 and ΔC = 1 %.

		Generation							
Mating scheme	Variance	0	1	2	3	4	5	6	7
RM	V_A	0.208	0.163	0.231	0.265	0.188	0.260	0.261	0.225
PAM	V_A	0.167	0.232	0.213	0.201	0.215	0.217	0.267	0.281
PAMCM	V_A	0.208	0.200	0.210	0.245	0.279	0.243	0.363	0.340
MCM1	V_A	0.195	0.162	0.244	0.263	0.248	0.244	0.254	0.227
MCM2	V_A	0.203	0.241	0.272	0.197	0.215	0.253	0.254	0.223
MCM3	V_A	0.206	0.194	0.259	0.282	0.279	0.242	0.232	0.177
MCM4	V_A	0.199	0.188	0.252	0.255	0.266	0.262	0.228	0.232
RM	V_E	0.271	0.270	0.227	0.267	0.199	0.218	0.230	0.229
PAM	V_E	0.247	0.246	0.241	0.292	0.248	0.223	0.272	0.247
PAMCM	V_E	0.214	0.217	0.235	0.228	0.316	0.205	0.279	0.217
MCM1	V_E	0.243	0.201	0.250	0.253	0.231	0.267	0.301	0.241
MCM2	V_E	0.272	0.294	0.153	0.154	0.239	0.203	0.256	0.213
MCM3	V_E	0.225	0.228	0.198	0.213	0.198	0.227	0.258	0.232
MCM4	V_E	0.226	0.247	0.245	0.195	0.251	0.200	0.228	0.195
