# Supplementary material for: Optimization of selection contribution and mate allocations in monoecious tree breeding populations
Source: BMC Genet. 2009 Nov 6;10:70. doi: 10.1186/1471-2156-10-70 (PMC2776599; doi:10.1186/1471-2156-10-70)
Supplement: Additional file 2 — Object functions for the various mating schemes. This section contains a full list of object functions used throughout the study. [file 1471-2156-10-70-S2.pdf]

Object functions for the various mating schemes

Throughout the study, the loss function, $L(\mathbf{X})$, was subjected to attain the contribution unit of all trees as follows

$$\sum_{j=1}^{n}[\mathbf{X}(i,j)+\mathbf{X}(j,i)]=\zeta_i,$$

where n is the number of selected trees, $\mathbf{X}$ is the mate allocation matrix and ζ_i is the contribution units of tree i.

The object functions used were as follows:

RM –

$$L(\mathbf{X})=constant,$$

where the constant was large enough so that all suggested states were accepted.

PAM –

$$L(\mathbf{X})=\sum_{i=1}^{n}\sum_{j=1}^{n}\mathbf{B}(i,j)\mathbf{X}(i,j)+\frac{\sum_{i=1}^{n}\sum_{j=1}^{n}[\boldsymbol{X}(i,j)]^2-\left[\sum_{i=1}^{n}\sum_{j=1}^{n}\boldsymbol{X}(i,j)\right]^2/n}{n-1},$$

where $\mathbf{B}(i,j)=\left|b_i-b_j\right|$ is the difference in EBV between pairs of mates of trees i and j.

PAMCM –

$$L(\mathbf{X})=\sum_{i=1}^{n}\sum_{j=1}^{n}\mathbf{B}(i,j)\mathbf{X}(i,j)+\frac{\sum_{i=1}^{n}\sum_{j=1}^{n}[\boldsymbol{B}(i,j)\boldsymbol{X}(i,j)]^2-\left[\sum_{i=1}^{n}\sum_{j=1}^{n}\boldsymbol{B}(i,j)\boldsymbol{X}(i,j)\right]^2/n}{n-1}.$$

MCM1 –

$$L(\mathbf{X})=\sum_{i=1}^{n}\sum_{j=1}^{n}\mathbf{A}(i,j)\mathbf{X}(i,j),$$

where $\mathbf{A}$ is the additive relationship matrix of the selected cohort of trees.

MCM2 –

$$L(\mathbf{X}) = \sum_{i=1}^{n}\sum_{j=1}^{n}\mathbf{A}(i,j)\mathbf{X}(i,j) + \frac{\sum_{i=1}^{n}\sum_{j=1}^{n}[\boldsymbol{X}(i,j)]^2 - \left[\sum_{i=1}^{n}\sum_{j=1}^{n}\boldsymbol{X}(i,j)\right]^2/n}{n-1}.$$

MCM3 –

$$L(\mathbf{X}) = \sum_{i=1}^{n}\sum_{j=1}^{n}\mathbf{A}(i,j)\mathbf{X}(i,j) + \sum_{i=1}^{n}\sum_{j=1}^{n}\mathbf{X}(i,j)\mathbf{X}(i,j).$$

MCM4 -

$$L(\mathbf{X}) = \frac{\sum_{i=1}^{n}\sum_{j=1}^{n}[\boldsymbol{B}(i,j)\boldsymbol{X}(i,j)]^2 - \left[\sum_{i=1}^{n}\sum_{j=1}^{n}\boldsymbol{B}(i,j)\boldsymbol{X}(i,j)\right]^2/n}{n-1} + \frac{\sum_{i=1}^{n}\sum_{j=1}^{n}[\boldsymbol{X}(i,j)]^2 - \left[\sum_{i=1}^{n}\sum_{j=1}^{n}\boldsymbol{X}(i,j)\right]^2/n}{n-1}.$$
